# Decoding the Gut Microbiota–Gestational Diabetes Link: Insights from the Last Seven Years

**DOI:** 10.3390/microorganisms12061070

**Published:** 2024-05-25

**Authors:** Luis Ricardo Balleza-Alejandri, Emiliano Peña-Durán, Alberto Beltrán-Ramírez, Africa Samantha Reynoso-Roa, Luis Daniel Sánchez-Abundis, Jesús Jonathan García-Galindo, Daniel Osmar Suárez-Rico

**Affiliations:** 1 Doctorado en Farmacología, Centro Universitario de Ciencias de la Salud, Universidad de Guadalajara, Guadalajara 44340, Mexico; luis.balleza3286@alumnos.udg.mx (L.R.B.-A.); africa.reynoso0835@alumnos.udg.mx (A.S.R.-R.); 2 Licenciatura en Médico Cirujano y Partero, Centro Universitario de Ciencias de la Salud (CUCS), Universidad de Guadalajara, Guadalajara 44340, Mexico; emilianodupe@live.com; 3 Departamento de Fisiología, Centro Universitario de Ciencias de la Salud, Universidad de Guadalajara, Calle Sierra Mojada 950, Independencia Oriente, Guadalajara 44340, Mexico; dralbertobeltran@gmail.com (A.B.-R.); dr.jonathangarcia1418@gmail.com (J.J.G.-G.); 4 Hospital Civil de Guadalajara, Fray Antonio Alcalde, Instituto de Patología Infecciosa y Experimental, Guadalajara 44200, Mexico; daniels.abundis@gmail.com

**Keywords:** microbiota, diabetes, gestational diabetes, probiotics, drugs

## Abstract

The human microbiome, a complex ecosystem of bacteria, viruses, and protozoans living in symbiosis with the host, plays a crucial role in human health, influencing everything from metabolism to immune function. Dysbiosis, or an imbalance in this ecosystem, has been linked to various health issues, including diabetes and gestational diabetes (GD). In diabetes, dysbiosis affects the function of adipose tissue, leading to the release of adipokines and cytokines, which increase inflammation and insulin resistance. During pregnancy, changes to the microbiome can exacerbate glucose intolerance, a common feature of GD. Over the past years, burgeoning insights into the gut microbiota have unveiled its pivotal role in human health. This article comprehensively reviews literature from the last seven years, highlighting the association between gut microbiota dysbiosis and GD, as well as the metabolism of antidiabetic drugs and the potential influences of diet and probiotics. The underlying pathophysiological mechanisms discussed include the impact of dysbiosis on systemic inflammation and the interplay with genetic and environmental factors. By focusing on recent studies, the importance of considering microbial health in the prevention and treatment of GD is emphasized, providing insights into future research directions and clinical applications to improve maternal–infant health outcomes.

## 1. Introduction

Recent research underscores the intricate relationship between the human gut microbiota and gestational diabetes (GD), a condition that affects a significant portion of pregnancies worldwide. The human microbiome, particularly the gut microbiota, plays a pivotal role in various metabolic functions, including glucose and lipid homeostasis. Alterations in the gut microbiota, known as dysbiosis, have been implicated in the pathogenesis of several metabolic diseases, including obesity, type 2 diabetes (T2D), and GD. GD not only complicates pregnancy but also poses long-term health risks for both the mother and the offspring [1].

Pregnancy induces significant changes in gut microbiota composition, which may influence the mother’s glucose metabolism and contribute to the development of GD. Research indicates that gut microbiota composition during pregnancy differs between women with GD and healthy pregnant women, suggesting potential targets for therapeutic interventions. Furthermore, the interaction between GD and gut microbiota composition extends to the offspring, affecting their metabolic pathways and possibly predisposing them to metabolic diseases later in life [2,3].

### 1.1. The Human Microbiome

The human microbiome plays a critical role in metabolic regulation, immune function, and behavior, comprising over 100 billion cells (mostly in the gastrointestinal tract) [2] and containing 27 times more genes than the human genome [3,4]. The microbiota is defined as a complex ecosystem of microorganisms, including bacteria, viruses, and protozoa, living in different areas of the body. Over 70% of the microbiota resides in the gastrointestinal tract in a mutually beneficial relationship with its host. The microbiota plays a significant role in many metabolic functions, including the modulation of glucose and lipid homeostasis, the regulation of satiety, and the production of energy and vitamins. There is growing evidence that any modification in the composition of the microbiota can lead to various diseases, including cardiometabolic diseases. This is because alterations in the composition of the microbiota can cause insulin resistance, inflammation, and vascular and metabolic disorders. For instance, dysbiosis or imbalance in the microbiota can lead to a chronic low-grade inflammatory state, which in turn can promote insulin resistance, a precursor to diabetes [1].

### 1.2. Gut Microbiota Dysbiosis in Diabetes

Diabetes is a heterogeneous group of metabolic diseases characterized by hyperglycemia, which may result in long-term complications leading to damage to many of the body’s systems, especially kidneys, nerves, eyes, and blood vessels [5].

Type 1 diabetes (T1D) is primarily an autoimmune condition whereby the immune system mistakenly attacks and destroys the insulin-producing β-cells in the pancreas. Recent research highlights the influence of genetic, epigenetic, and environmental factors on the development of T1D, with a significant focus on the role of the gut microbiota. Alterations in this gut microbiota, due to dietary changes, antibiotic use, or infections, can lead to dysbiosis, contributing to autoimmune and inflammatory diseases, showing that the gut microbiota composition in T1D patients differs significantly from healthy individuals, suggesting a link between gut health and the development of T1D [6].

Type 2 diabetes (T2D), a prevalent metabolic disorder globally, arises mainly due to inadequate insulin secretion from pancreatic β-cells and the reduced responsiveness of insulin-sensitive tissues [7]. Emerging research highlights the significant role of gut microbiota dysregulation alongside adipokine imbalance and inflammation in its pathogenesis [8]. In patients with T2D, dysbiosis has been observed. This dysbiosis is characterized by a reduced overall diversity and richness of the gut microbiota. Specific bacterial taxa such as *Granulicatella* and *Prevotella* have been associated with an increased risk of developing T2D. Some differences include higher abundances of *Escherichia, Shigella*, and *Collinsella*, among others [9]. Unlike healthy controls who exhibit daily oscillations in numerous operational taxonomic units, T2D patients show an arrhythmic microbiota lacking diversity in *Bacteroidetes* and *Firmicutes*. This dysbiosis in the gut microbiome of T2D patients underscores the intricate relationship between metabolic health and gut microbiota composition [10]. Through these mechanisms, the gut microbiota emerges as a pivotal factor in the development and progression of T2D, offering promising avenues for future research aimed at restoring gut microbiota balance and function.

**Figure 1:**
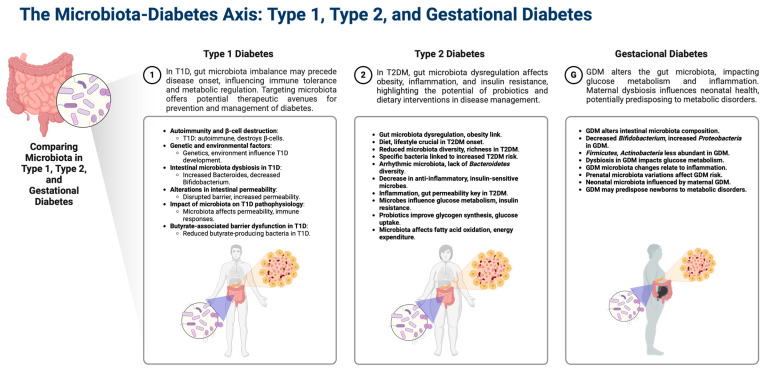
Comparative overview of the gut microbiota in Type 1, Type 2, and gestational diabetes (GD). The diagram illustrates distinctive microbial profiles associated with each diabetes type and their potential consequences on host metabolism and immune responses, emphasizing the microbiota’s role in disease mechanisms and therapeutic opportunities. This figure was created with BioRender software (https://biorender.com/, accessed on 18 March 2024).

The dysbiosis characteristic of T2D, marked by a reduced diversity and changes in specific bacterial taxa, is visually summarized in Figure 1, illustrating the unique microbial profiles associated with each type of diabetes and their potential consequences on host metabolism and immune responses.

### 1.3. Gestational Diabetes

Gestational diabetes (GD) is defined as the dysregulation of glucose tolerance that initiates or occurs in pregnant women [11]. The International Association of Diabetes and Pregnancy Study Group (IADPSG) recommends that all women in the gestational stage maintain the following glucose goals: fasting glucose <95 mg/dL and either 1 h postprandial glucose <140 mg/dL or 2 h postprandial glucose <120 mg/dL [12]. The American College of Obstetricians and Gynecologists (ACOG) recommends fasting glucose of 70–95 mg/dL, glucose at 1 h postprandial 110–140 mg/dl, and glucose at 2 h postprandial 100–120 mg/dL. Among these criteria, those of the IADPSG currently have the highest diagnostic capability for GD [13].

The pathophysiological mechanisms of the disease described so far explore different pathways, of which the following stand out: (1) dysfunction of beta cells caused by the hyperglycemic state secondary to physiological changes of pregnancy (such as insulin sensitivity adaptation). In the early stage of pregnancy, its sensitivity increases, promoting energy reserves within adipose tissue in preparation for gestation; these changes promote insulin resistance. (2) Insulin resistance, which causes inadequate translocation of glucose transporter 4 (GLUT4), through reduced tyrosine phosphorylation and increased threonine/serine in the insulin receptor [14]. (3) Studies have found differences in gut microbiota composition between healthy and GD-affected pregnant women, including variations in microbial diversity and specific bacterial abundances [15]. These findings suggest the gut microbiota’s potential as a biomarker for early detection of GD and a target for interventions to reduce GD risk. Moreover, alterations in the gut microbiota may be associated with GD’s metabolic dysregulations, including glucose intolerance and insulin resistance. The role of the gut microbiota in GD underscores the importance of considering microbial health in managing and preventing this condition.

### 1.4. Pregnancy and Its Impact on Gut Microbiota Composition

The microbiota, present not only in the gastrointestinal tract but also in the oral cavity, skin, lungs, and genitourinary tract, and even intrauterine in the placenta, previously considered sterile [16], undergoes modifications during pregnancy.

Pregnancy is a unique physiological state that brings temporary changes in physical structure, hormonal levels, metabolism, and the immune system of a woman [2,4]. These changes are vital for maintaining a stable state between mother and fetus, influencing the composition of microorganisms in various areas of the pregnant woman’s body [3,4]. Moreover, physiological, hormonal, and dietary changes linked to this state also impact the likelihood of developing conditions [3,4,17,18]. Variations are observed across different trimesters of pregnancy, with a shift in the intestinal microbiota composition from the first to the second trimester [17]. At least 1800 genera and approximately 15,000 to 36,000 species of bacteria, of which 94–98% belong to four phyla (*Firmicutes* (64%), *Bacteroidetes* (23%), *Proteobacteria* (8%), and *Actinobacteria* (3%) [17,19]), specifically show an increase in the *Firmicutes*/*Bacteroidetes* ratio, among others [20]. Such changes are linked to insulin resistance and the development of GD in up to 10% of patients, which normalizes after delivery.

Temporal changes in the vaginal microbiome associated with term pregnancies have been identified, with a more homogeneous microbiome dominated by *Lactobacillus* emerging in the second trimester [19,21]. Furthermore, an increased abundance of various taxa, including *Sneathia amnii*, *Prevotella clades*, *Lachnospiraceae*, and *Saccharibacteria* (associated with low levels of vitamin D), has been linked to the risk of preterm birth [21].

Regarding the placental microbiota, Ruiz-Treviño et al. (2023) note that the placenta contains a microbiome of low abundance but high metabolic activity, mainly composed of non-pathogenic commensal microbiota from the phyla *Firmicutes*, *Tenericutes*, *Proteobacteria*, *Bacteroidetes*, and *Fusobacteria* [22]. The placental microbiome profiles showed significant similarities with the oral microbiome of non-pregnant individuals. Sequence-based operational taxonomic unit analyses indicated a correlation between the placental microbiome and a history of long-term prenatal infection, as well as with preterm births before 37 weeks.

### 1.5. Drugs Used in Pregnancy Can Affect the Microbiota 

Gut microbiota has an essential and wide role in the physiology of the human body, ranging from modulation of immune aspects to drug metabolism [23]. Several conditions, such environmental factors, diseases, drug intake, lifestyle, and even pregnancy, can be associated with gut microbiota alterations [24,25].

Regarding drugs, these alterations can be described as a bidirectional interaction, meaning that the drug response can be altered by the gut microbiota and the drug can modify the environment related to gut microbiota [26]. Some drug families have been described to modify this environment, including antibiotics, proton pump inhibitors, selective serotonin reuptake inhibitors, angiotensin-converting-enzyme inhibitors (ACEIs), angiotensin-II-receptor antagonists (ARA-II), antihistamines, opiates, statins, oral contraceptives, paracetamol, and metformin [23,26].

During pregnancy, changes in the microbiota can occur before the onset of GD, and this must be considered carefully due to the impact on the maternal and neonatal health [17]. Metagenomics studies have shown that during pregnancy, *Prevotella*, *Ruminococcaceae*, and *Parabacteroides distasonis* increase. Even though insulin has been the regular therapy for GD, treatment based on metformin, diet, and lifestyle changes evidenced improvement in glucose levels [27,28,29]. This metformin-based treatment showed a reduction in *Firmicutes* and *Peptoestreptococcaceae*, as well as an increase in *Proteobacteria*, *Enterobacteriaceae*, and *Coprococcus catus*, which were correlated with the mean postprandial glycemia, Body mass index (BMI) and weight increase [7].

### 1.6. The Microbiota and Its Intervention in the Metabolism of Antidiabetic Drugs

**Figure 2:**
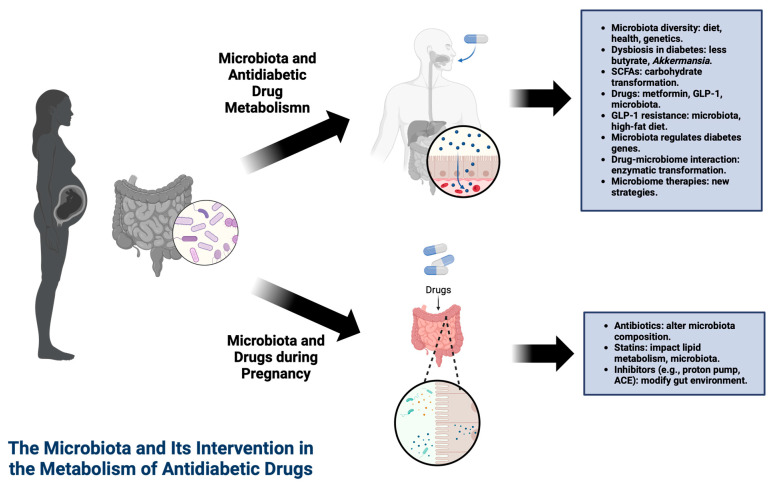
Interplay between the gut microbiota and antidiabetic drug metabolism in pregnant women. This illustration depicts the influence of diet, health, genetics, and medications such as metformin and GLP-1 on microbiota diversity and function. It also highlights the potential resistance mechanisms to GLP-1 and the therapeutic interventions targeting the microbiome. This figure was created with BioRender software (https://biorender.com/, accessed on 18 March 2024).

The composition and diversity of the intestinal microbiota are subject to the influence of various factors, such as diet, host health, age, ethnicity, and genetics, leading to significant variations among individuals (Figure 2) [3,30,31]. Obesity and type 2 diabetes cause dysbiosis in the intestinal microbiota, affecting the abundance of butyrate-producing bacteria and *Akkermansia muciniphila*, the latter considered a biomarker for glucose intolerance [30]. Thus, the key role of the intestinal microbiota in regulating host metabolism and the associations of intestinal microbial dysbiosis with the development of obesity and diabetes [32] have been extensively explored, serving as a mediating pathway for the therapeutic effects of antidiabetic medications [26,31,32].

The intestinal microbiota plays a crucial role in transforming non-digestible carbohydrates into short-chain fatty acids (SCFAs), such as propionate, acetate, and butyrate, which in turn positively affect the action of hypoglycemic agents in type 2 diabetes [19,30]. It has been observed that hypoglycemic agents can alter the intestinal microbiota, improving glucose metabolism and energy balance [26,32,33]. Additionally, the intestinal microbiota can influence the efficacy of orally ingested drugs, as microbiota enzymes can metabolize xenobiotics and affect the pharmacogenetics of medications [30,34]. Supplementation with probiotic strains and nutraceuticals has also shown health benefits in obesity and related diseases, in both animal and human studies [26,31,35]. For example, metabolites mediated by SGLT-1 produced by *Lactobacillus* result in increased glucose absorption in Caco-2 cells, aiding in the modulation of metformin’s glucose effects [36]. Gu et al. (2017) found that differential therapeutic responses are related to distinct abilities of microbial communities in two microbiome groups to metabolize bile acids (BA) driven by *Bacteroides*, compared to those belonging to a group composed predominantly of *Prevotella* [37].

Grasset et al. observed that GLP-1-induced insulin secretion and gastric emptying were disrupted in mice fed a high-fat diet, noting a deregulated intestinal microbiota that mechanically damaged the GLP-1-activated gut–brain axis, thus leading to GLP-1 resistance [38]. Translating these results, it is possible that a deregulated microbiota could lead to resistance to GLP-1 agonist antidiabetics. Furthermore, intestinal microbes have been shown to contribute to the efficacy and safety of drugs by enzymatically transforming the structure of drugs and altering their bioavailability, bioactivity, or toxicity [34,39,40]. For example, Wang et al. (2023) found that the intestinal microbial isoenzyme Dau-d4 (a derivative of daurisoline), which selectively inhibits the activity of mDPP4 compared to hDPP4, increases active GLP-1 levels and improves glucose metabolism in diabetic mice, suggesting that variations in microbial DPP4 activities could possibly contribute to the heterogeneous responses to sitagliptin observed among patients with type 2 diabetes [41].

The composition of the intestinal microbiota also influences the regulation of various genes in T2D. Although reports are limited, they suggest a complex interaction between genes and microbes in the etiology of the disease, in addition to playing a fundamental role in the epigenetic regulation of genes by modifying DNA methylation [31]. The interaction between the human intestinal microbiome, drugs, and related xenobiotics is extremely complex and must be considered bidirectional. Based on the aforementioned connections between the intestinal microbiota and the therapeutic effects of antidiabetics, there arises the possibility of identifying new therapeutic intervention modalities aimed at the intestinal microbiome, selecting the best medication strategies, and predicting the efficacy of antidiabetic drugs.

### 1.7. Use of Probiotics in Gestational Diabetes

Recently, probiotic supplementation has emerged as a potential strategy to mitigate some GD-associated risks, though studies have shown varied results. In this review, we examine the recent literature on the effects of probiotics on GD and its potential mechanisms of action. A systematic review and meta-analysis by Masulli et al. (2020) found a minor but statistically significant reduction in fasting plasma glucose, but not in the incidence of GD, suggesting that probiotics may moderately impact glycemic parameters, but their efficacy in preventing GD is limited [42]. Conversely, Davidson et al. (2021) highlighted an increased risk of pre-eclampsia associated with probiotic administration compared to placebo. This finding indicates a potential adverse effect of probiotics, emphasizing the need for caution and further investigation into the safety profile of probiotic supplementation during pregnancy [43].

Pakmehr et al. (2022) focused on the preventive effects of probiotics on GD occurrence. The study concluded that while probiotics might reduce the incidence of GD, the evidence is not conclusive, and no significant benefits or harms related to probiotic supplements on secondary outcomes were observed [44]. Shahriari et al. (2021) investigated the effects of probiotic supplementation from the early second trimester until the 24th week of pregnancy. The study found no significant reduction in the risk of GD or improvement in other neonatal and maternal outcomes, questioning the efficacy of probiotics in preventing GD [45]. The reviewed studies present a complex picture of the role of probiotics in managing GD. While there is some evidence to suggest that probiotics can marginally improve fasting plasma glucose (FPG) levels, their impact on reducing the incidence of GD is not substantial. Moreover, the potential risk of pre-eclampsia associated with probiotic use underscores the importance of a cautious approach to their application in pregnant populations. The variability in study outcomes highlights the need for personalized considerations, further research, and a deeper understanding of the mechanisms through which probiotics influence maternal and fetal health. Probiotic supplementation during pregnancy presents a potentially beneficial but complex intervention for managing GD. Although minor improvements in metabolic parameters have been observed, the lack of significant impact on GD incidence and potential safety concerns, such as an increased risk of pre-eclampsia, necessitate further investigation. Future studies should aim to clarify the efficacy, safety, and mechanism of action of probiotics, guiding healthcare professionals in optimizing maternal and neonatal health outcomes.

### 1.8. Dietary Influences on Gut Microbiota and the Risk of Gestational Diabetes

The interplay between diet, gut microbiota, and GD is critical area of active research, with profound implications for maternal and infant health. Diet patterns notably influences gut microbiota composition and function, thereby, impacting the development and progression of GD. A vegetarian diet in early pregnancy is associated with a distinct gut microbiota composition, suggesting diet type can influence gut microbiota during gestational periods [46]. Diets rich in complex carbohydrates and low in fats have been associated with higher levels of *Bifidobacteria* in the maternal microbiome, which in turn enhances infant gut microbiome diversity [47]. This may offer a protective role against obesity and aid in the development of the immune system. Moreover, dietary interventions such as the inclusion of fish oil and probiotics could influence gut microbiota composition, although specific bacterial species have been directly linked to the onset of GD [48].

Significant alterations in the gut microbiota, including shifts among the phyla *Firmicutes*, *Bacteroidetes*, and *Actinobacteria*, are observed in women with GD and may extend postpartum, potentially affecting the newborn’s development [49,50]. Additionally, probiotic supplementation early in pregnancy has been correlated with reduced GD incidence, highlighting the need for further research to verify these findings and explore their long-term effects [51]. Short-term dietary interventions can modify the *Firmicutes*/*Bacteroidetes* ratio and specific gut taxa, impacting metabolic parameters like blood glucose levels and body mass index [52].

The gut mycobiome, encompassing fungi such as *Hanseniaspora* and *Candida*, also plays a role in metabolic homeostasis in GD and can be altered through dietary means [53]. Despite the presence of GD, the overall composition of the gut microbiota may not significantly differ from healthy pregnant women, yet certain bacterial genera may show associations with glucose metabolism [54].

Dietary choices during pregnancy significantly affect the gut microbiota in women with GD and their infants. A diet high in complex carbohydrates may foster a more favorable maternal and infant microbiome, offering potential protection against metabolic and immune-related conditions. Probiotics and specific dietary interventions also show promise in modulating the gut microbiota and reducing GD risk. However, specific bacterial species alone do not appear to predict the onset of GD. The emerging role of the gut mycobiome in GD highlights the potential of diet interventions to influence fungal populations and metabolic health, underscoring the importance of dietary management in the prevention and treatment of GD, with implications for both maternal and neonatal health.

### 1.9. Types of Dysbiosis in Gestational Diabetes

Pregnancy itself induces significant changes in the gut microbiome, affecting various bacterial populations from the first trimester; *Bifidobacterium* [55], *Proteobacteria*, and *Actinobacteria* increase during pregnancy, while populations of *Faecalibacterium* decrease [49]. While the precise mechanisms behind these alterations are not fully described, they are suggested to result from the radical change in the hormonal profile typical of gestation. While some alterations may lead to pathological states, others are suggested to be beneficial and necessary for fetal growth [55].

In the context of GD, a large number of studies provide evidence on the changes in the gut microbiota accompanying this metabolic alteration. Different studies have explored and identified that populations of various bacterial species increase or decrease during GD. Chen et al. described that species of the genus *Firmicutes* and *Actinobacteria* are less abundant, while those of the genus *Bacteroidetes* increase excessively [56]; similar results were found by Li et al. when comparing fecal microbiota between women with GD and normoglycemic women [57]. Liu et al. described a decrease in gut microbiota richness in GD, specifically, a decrease in *Bacteroides* and *Akkermansia*, and an increased abundance of *Faecalibacterium* [58]. *Gammaproteobacteria* and *Hemophilus* populations also abound in GD [59], as do *Ruminococcaceae* [60]. Wang et al. found abundant populations of *Lachnospiraceae* but a scarcity of *Enterobacteriaceae* and *Ruminococcaceae*, results that differ from those obtained by other authors [18].

It has been proposed that the microbiota not only coincides with GD but also plays a relevant role in its pathogenesis, as suggested by the findings of Crusell et al., who, when comparing the intestinal bacterial profile of women with GD and women with T2D without pregnancy, found in both an excess of colonies of bacteria from the genera *Collinsella*, *Desulfovibrio*, and *Blautia* [61]. Various studies have shown that certain changes in bacterial colonies are related to specific mechanisms associated with the pathogenesis of GD; *Faecalibacterium*, *Sutterella*, *Collinsella*, and *Blautia* were associated with alterations in fasting glucose, insulin, the Homeostasis Model Assessment of Insulin Resistance (HOMA-IR) index, and C-reactive protein (CRP) levels, respectively [62]. Liu et al. also associated the abundance of *Faecalibacterium* with a higher production of inflammatory factors and the presence of hyperglycemia [58], while Crusell et al. correlated a higher number of *Christensenella* with increased fasting glucose and *Akkermansia* with decreased insulin sensitivity [63].

Numerous studies have identified that the microbiota in GD also varies throughout pregnancy. Zheng et al. observed that, during the first trimester, *Acinetobacter*, *Bacteroides*, *Parabacteroides*, *Sphingomonas*, *Streptophyta*, *Holdemania*, *Haemophilus*, *Erysipelotrichaceae*, and various species of *Clostridium* are more abundant. In contrast, during the second trimester (T2), *Blautia*, *Rothia*, *Bilophila*, *Bifidobacterium*, *Anaerococcus*, *Deltaproteobacteria*, and *Firmicutes* increase [20]. Finally, during the third trimester, *Bacteroidetes*, *Proteobacteria*, *Faecalibacterium*, *Anaerotruncus*, *Collinsella*, and *Rothia* abound, while the populations of *Firmicutes* and *Veillonella* decrease [61,64]. Similarly, it has been reported that the abundance of bacteria from the *Ruminoccaceae* family is directly related to the development of GD in late stages of pregnancy [60].

### 1.10. Effects on the Fetus

The bacterial colonization of the intestine is vitally important for the development and maturation of the offspring’s metabolic pathways; this occurs mainly through vertical transmission of maternal microbiota or during breastfeeding. Significant changes in the intestine, such as those occurring during GD, also impact the varieties of bacteria that can be found in the offspring [65]. It has been described that the intestinal flora of newborns from mothers with GD have a greater abundance of *Firmicutes* and *Turicibacter*, while the populations of *Proteobacteria*, *Veillonella*, *Megasphaera*, *Prevotella*, *Bacteroidetes*, *Rothia*, and *Actinobacteria* are impoverished [63]. Su et al. conducted a similar experiment and found an increased abundance in *Proteobacteria* and *Actinobacteria*, while *Firmicutes*, *Bacteroidetes*, *Synergistetes*, *Thermi*, *Spirochaetes*, *Chloroflexi*, and *Euryarchaeota* decreased compared to the healthy control group [50]. In feces of newborns from mothers with GD, Ponzo et al. found a greater relative abundance of *Bifidobacterium*, *Streptococcus*, *Staphylococcus*, *Escherichia*, and *Enterococcaceae*. They also described that infants who are breastfed have a fecal microbiota with a greater abundance of *Actinobacteria* and *Proteobacteria* than those fed formula [66].

Some changes in the microbiota of neonates from pregnancies with GD affect bacterial populations that are fundamental for the development of neonatal immunity. Soderborg et al. analyzed fecal samples from newborns and found decreased colonies of *Lactobacillus*, *Flavonifractor*, *Erysipelotrichaceae*, and *Gammaproteobacteria*, bacteria that fulfill the mentioned function. At the same time, *Phascolarctobacterium*, a microorganism associated with the suppression of early immune system functions, was found in increased proportions [52]. These changes may predispose the newborn to inflammatory and metabolic changes in later stages of extrauterine life.

### 1.11. Future Perspectives

The burgeoning research into the gut microbiota’s influence on gestational diabetes (GD) suggests several promising directions for future investigations. The dynamic shifts in microbial communities throughout pregnancy present a pivotal area for longitudinal studies, which could provide insights into how changes in the microbiota relate to the development and progression of GD, potentially unveiling new markers for early detection [58]. Furthermore, the connections between the placental microbiome and the oral microbiome of non-pregnant individuals hint at intriguing mechanisms of microbial transmission and colonization that merit further exploration. Such studies could reveal critical pathways through which maternal microbiota impacts fetal health and the genesis of metabolic diseases [20]. Clinically, the modulation of gut microbiota emerges as a viable strategy for managing and preventing GD. Innovations in probiotic formulations and dietary adjustments hold the potential to rebalance gut microbiota, thereby mitigating GD risks. Developing precise microbiota signatures that predict GD could lead to breakthroughs in preemptive healthcare, enabling interventions before the onset of disease symptoms [42]. However, the field must navigate several challenges to harness the full potential of these discoveries. Establishing the causality between microbiota alterations and GD, standardizing research methodologies, and ensuring the safety of microbiota-targeted interventions in pregnant women are critical hurdles that need addressing. As the research community moves forward, tackling these limitations will be essential for translating microbial insights into effective clinical practices [43].

## 2. Conclusions

The exploration of the gut microbiota’s role in GD has opened new horizons for understanding this complex condition. As we deepen our knowledge of the microbiome’s impact on metabolic health during pregnancy, the potential to develop novel diagnostics and therapeutic interventions grows. Future research should aim to unravel the causal mechanisms, optimize microbiota-based treatments, and establish tailored nutritional and lifestyle recommendations to mitigate GD risk, ensuring healthier outcomes for both mothers and their offspring.

## Data Availability

Not applicable.
